# Effect of Dietary Lead on Intestinal Nutrient Transporters mRNA Expression in Broiler Chickens

**DOI:** 10.1155/2015/149745

**Published:** 2015-01-28

**Authors:** Roohollah Ebrahimi, Mohammad Faseleh Jahromi, Juan Boo Liang, Abdoreza Soleimani Farjam, Parisa Shokryazdan, Zulkifli Idrus

**Affiliations:** ^1^Institute of Tropical Agriculture, Universiti Putra Malaysia, 43400 Serdang, Selangor, Malaysia; ^2^Faculty of Veterinary Medicine, Universiti Putra Malaysia, 43400 Serdang, Selangor, Malaysia

## Abstract

Lead- (Pb-) induced oxidative stress is known to suppress growth performance and feed efficiency in broiler chickens. In an attempt to describe the specific underlying mechanisms of such phenomenon we carried out the current study. Ninety-six one-day-old broiler chicks were randomly assigned to 2 dietary treatment groups of 6 pen replicates, namely, (i) basal diet containing no lead supplement (control) and (ii) basal diet containing 200 mg lead acetate/kg of diet. Following 3 weeks of experimental period, jejunum samples were collected to examine the changes in gene expression of several nutrient transporters, antioxidant enzymes, and heat shock protein 70 (Hsp70) using quantitative real-time PCR. The results showed that addition of lead significantly decreased feed intake, body weight gain, and feed efficiency. Moreover, with the exception of GLUT5, the expression of all sugar, peptide, and amino acid transporters was significantly downregulated in the birds under Pb induced oxidative stress. Exposure to Pb also upregulated the antioxidant enzymes gene expression together with the downregulation of glutathione S-transferase and Hsp70. In conclusion, it appears that Pb-induced oxidative stress adversely suppresses feed efficiency and growth performance in chicken and the possible underlying mechanism for such phenomenon is downregulation of major nutrient transporter genes in small intestine.

## 1. Introduction

Lead (Pb) is one of the widespread environmental pollutants that induce a broad range of physiological and biochemical dysfunctions in animals [1]. Mahesar et al. [2] reported that most of the poultry feed samples which they analyzed contained greater amount of Pb and cadmium (Cd) than the maximum tolerable levels for poultry. This observation suggests the possibility of accumulation of Pb in commercially produced poultry meat and meat products. Being a cumulative poison, Pb is also accumulated in various organs, such as brain, liver, kidney, bone, and hemopoietic system [3–5] and inhibits growth [6, 7]. Several researchers suggested that the biochemical and molecular mechanism of Pb toxicity involve the induction of oxidative stress in target cells and generation of reactive oxygen species (ROS), followed by DNA damage and apoptosis partly via the (1) direct effect of Pb on cell membranes, (2) Pb-hemoglobin interactions, (3) consistent reduction in blood delta-aminolevulinic acid dehydratase (ALAD), an erythrocyte enzyme sensitive to Pb, and (4) effect of Pb on antioxidant defense systems of cells [8–13]. Low levels of intracellular ROS play a major role in redox signaling, but in high amounts ROS cause lipid peroxidation, compromise the cell membrane integrity, and deactivate the membrane-bound receptors and enzymes [14]. In addition, varying levels of ROS may impact mRNA and protein expression [15]. These changes are elicited primarily by transcriptional and posttranscriptional mechanisms via alterations in chromatin metabolism and transcription factors activity [16] and also RNA-binding proteins and microRNAs [17–19]. Evidently, Roche et al. [20] showed that increased production of ROS by gamma radiation in mice downregulates the mRNA abundance of intestinal sugar transporters. Nutrient transport is essential function of the small intestine and its disruption reduces nutrients utilization and feed efficiency. Regarding Pb-induced oxidative stress, except some reports on effects of Pb on loss of intestinal absorptive surface area [21, 22], there has not been a single study reporting on the alterations in mRNA expression of intestinal transporters. Earlier study of Damron et al. [21] demonstrated that feeding diet containing 1000 ppm Pb suppressed feed intake, body weight gain, and feed efficiency in chicken. The adverse effect of Pb on performance also has been reported for as low as 1 ppm [23, 24]. Decreased intestinal epithelial cell proliferation and loss of absorptive surface area under stress condition have been shown to be the reason for such poor performance [25]. However, Nasir et al. [26], Garriga et al. [27], and Hu and Guo [28] showed that oxidative stress by corticosterone or heat exposure increased glucose absorption, which might not be explained by the decreased small intestinal surface area and likely to be associated with a change of nutrient transporter in the small intestinal epithelium during stress. Some studies confirmed this possibility, showing that starvation stress increased expression of sodium glucose cotransporter 1 and peptide transporter 1 mRNA in the small intestine of chickens and rats [29–31].

To date, the actual mechanism by which ingestion of Pb suppresses body weight gain and feed efficiency remains unclear. We hypothesized that Pb affects mRNA expression of nutrient transporters across the intestinal wall and thereby reduces nutrients utilization. Therefore, the objective of this study was to determine the effects of Pb-induced oxidative stress on mRNA expression of 4 sugar (monosaccharides) transporters: Na^+^-dependent glucose and galactose transporter (SGLT1), glucose transporter (SGLT4), Na^+^-independent glucose, galactose and fructose transporter (GLUT2), and Na^+^-independent fructose transporter (GLUT5). Two other nutrient transporter genes, namely, oligopeptide transporter (PepT1) and the excitatory amino acid transporter (EAAT3), and antioxidant enzymes were also studied.

## 2. Materials and Methods

### 2.1. Animals

Ninety-six one-day-old (Cobb500) male broiler chicks were obtained from a local hatchery and weighed and housed at 12 battery cages of 8 chicks each. Feed and water were provided* ad libitum* and lighting was continuous. Experimental procedure followed the ACUC (Animal Care and Use Committee) of Universiti Putra Malaysia.

### 2.2. Experimental Design and Procedure

Commencing from day one, 6-replicate cages were randomly assigned to either (i) basal diet containing no supplemented lead acetate (control) (Table 1) or (ii) basal diet supplemented with 200 mg lead acetate/kg (Nacalai Tesque, Kyoto, Japan) [1, 32, 33]. The analyzed concentration of Pb in the control and lead supplemented diets was 0.155 (negligible) and 127 mg/kg, respectively. Body weight and feed intake were recorded and feed efficiency was calculated for the 1–21-day period. Accordingly, daily ingestion of Pb was 6 and 5100 *μ*g in control and lead supplemented birds, respectively. Analysis of liver samples confirmed Pb retention in birds tissue (0.05 and 0.64 mg/kg in control and lead supplemented birds, resp.).

At day 21, one bird from each cage was randomly selected and killed by cervical dislocation without previous starvation. Immediately, jejunum was removed and flushed with ice-cold saline. A 2 cm tissue section of mid jejunum was excised, frozen in liquid nitrogen, and stored at −80°C for further analysis.

### 2.3. mRNA Analysis

Total RNA was extracted from the jejunum samples (6 samples per treatment) using the RNeasy Midi Kit (Qiagen Inc., Valencia, CA) according to the manufacturer's protocol. Total RNA was quantified at 260/280 nm using the ND-1000 spectrophotometer (Nanodrop Technologies, Wilmington, DE) and stored at −80°C. The quality and integrity were also checked through agarose gel electrophoresis. The isolated RNA was subjected to two-step quantitative RT-PCR using the SYBR green assay with SYBR green PCR master mix (Applied Biosystems, Foster City, CA) using the ABI PRISM 7000 sequencing system (Applied Biosystems). Briefly, 10 *μ*g of total RNA was reverse-transcribed using Stratascript RT (Stratagene, La Jolla, CA) with oligo dT (5 *μ*g/*μ*L; IDT DNA, Coralville, IA) and random hexamers (5 *μ*g/*μ*L; IDT DNA). The cDNA (6.25 ng) was used in 25 *μ*L PCR reaction with final concentration of 0.25 ng/*μ*L. Glyceraldehyde phosphate dehydrogenase (GAPDH) was used as the endogenous control gene and primers were designed using the Primer3 software [34, 35] (Table 2). PCR was performed under the following conditions: 50°C for 2 min, 95°C for 10 min, and 40 cycles of 95°C for 15 s and 60°C for 1 min. A dissociation step consisting of 95°C for 15 s, 60°C for 30 s, and 95°C for 15 s was performed at the end of each PCR to verify amplification of a single product. The specificity of the amplification product was further verified by electrophoresis on a 0.8% agarose gel and by DNA sequencing. A real-time PCR was conducted for each primer pair in which cDNA samples were substituted with distilled H_2_O to verify that exogenous DNA was not present. Additionally, 1 *μ*g of RNA isolated by the procedure described above was substituted for cDNA in a real time PCR reaction to confirm that no genomic DNA contaminants were present in the RNA samples. Both of these negative controls showed no amplification after 40 cycles. The cycle numbers at which amplified DNA samples exceeded a computer-generated fluorescence threshold level were normalized and compared with determined relative gene expression. Higher cycle number values indicated lower initial concentrations of cDNA and thus lower levels of mRNA expression. Each sample was run in duplicate, and averaged duplicates were used to assign cycle threshold (CT) values. The ΔCT values were generated by subtracting experimental CT values from the CT values for GAPDH targets of each sample. The group with the highest mean ΔCT value (lowest gene expression) per amplified gene target was set to 0 and the mean ΔCT values of the other groups were set relative to this calibrator (ΔΔCT). The ΔΔCT values were calculated as powers of 2 (2^−ΔΔCT^) to account for the exponential doubling of the PCR [36, 37].

### 2.4. Statistical Analysis

The mRNA expression data were analyzed by 2-tailed *t*-test with unequal group variance using SAS statistical software [38]. The significance level was set at *P* < 0.05.

## 3. Results

**Figure 1:**
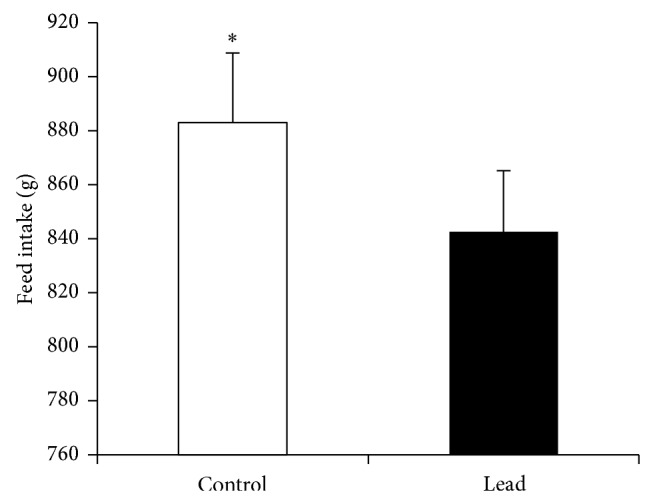
Effect of supplementation of Pb on feed intake in broiler chicken (1–21 days). Results are means of 6 replicates (*n* = 6). Error bar is standard deviation. ^*^ *P* < 0.05.

**Figure 2:**
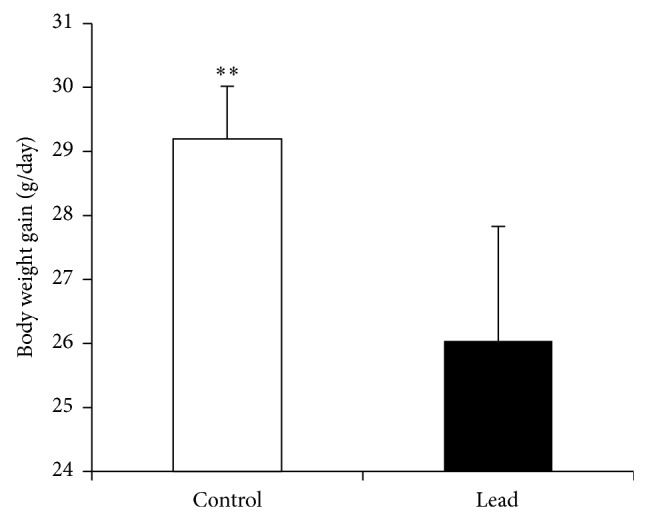
Effect of supplementation of Pb on body weight gain in broiler chicken. Results are means of 6 replicates (*n* = 6). Error bar is standard deviation. ^**^ *P* < 0.01.

**Figure 3:**
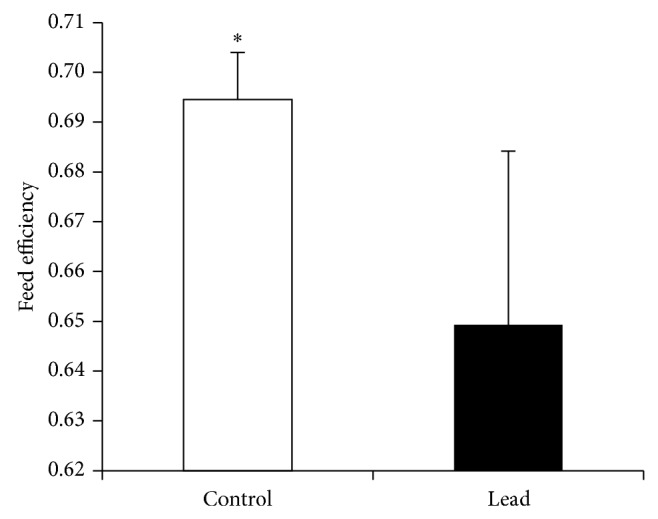
Effect of supplementation of Pb on feed efficiency in broiler chicken. Results are means of 6 replicates (*n* = 6). Error bar is standard deviation. ^*^ *P* < 0.05.

Exposure to Pb (200 mg Pb/kg diet) significantly suppressed feed intake (FI) (Figure 1), body weight gain (BWG) (Figure 2), and feed efficiency (Figure 3). Higher standard deviation in Pb-exposed birds may indicate the natural individual stress response variation.

**Figure 4:**
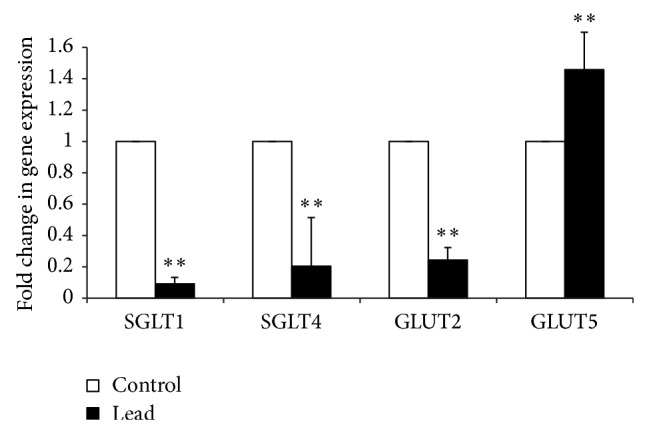
Effect of addition of Pb on relative mRNA abundance of SGLT1, SGLT4, GLUT2, and GLUT5 measured by real-time PCR in broiler chicken. Results are means of 6 replicates (*n* = 6). Error bar is standard deviation. ^**^ *P* < 0.01.

**Figure 5:**
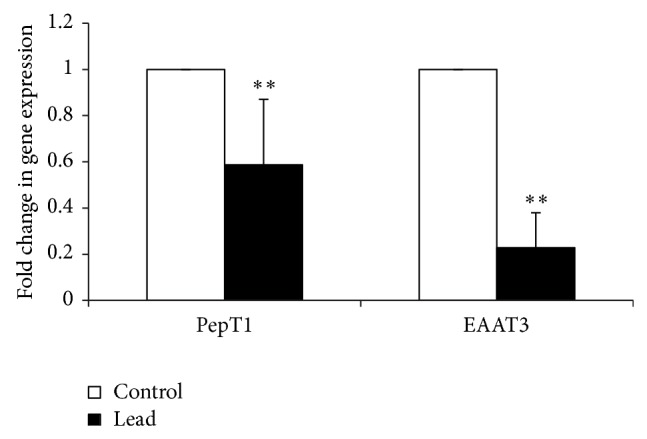
Effect of addition of Pb on relative mRNA abundance of PepT1 and EAAT3 measured by real-time PCR in broiler chicken. Results are means of 6 replicates (*n* = 6). Error bar is standard deviation. ^**^ *P* < 0.01.

The mRNA expressions of all studied sugar transporters except GLUT5 were significantly downregulated in Pb-exposed chicks (Figure 4). The mRNA expression of GLUT5 was upregulated in chicks exposed to Pb. A similar significant downregulation was observed in amino acid and peptide transporters mRNA expressions (Figure 5).

**Figure 6:**
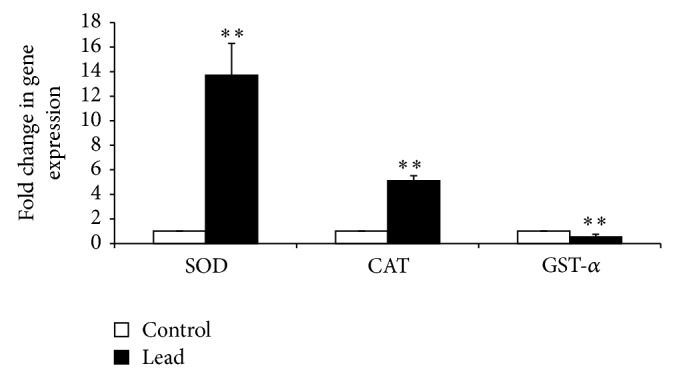
Effect of addition of Pb on relative mRNA abundance of antioxidant enzyme (SOD, CAT, and GST-*α*) measured by real-time PCR in broiler chicken. Results are means of 6 replicates (*n* = 6). Error bar is standard deviation. ^**^ *P* < 0.01.

**Figure 7:**
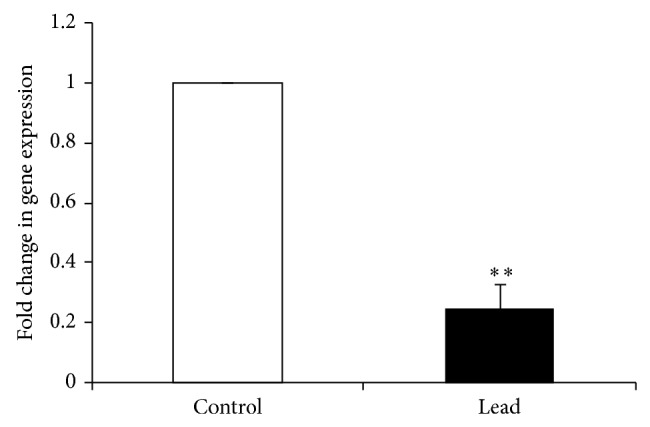
Effect of addition of Pb on relative mRNA abundance of Hsp70 measured by real-time PCR in broiler chicken. Results are means of 6 replicates (*n* = 6). Error bar is standard deviation. ^**^ *P* < 0.05.

Exposure to Pb significantly increased the major antioxidant enzymes, namely, superoxide dismutase (SOD) and catalase (CAT) mRNA expression (Figure 6), while decreasing the mRNA level of 3-glutathione S-transferase-*α* (GST-*α*) (Figure 6). Regarding the heat shock protein 70 (Hsp70), our results showed that intestinal Hsp70 mRNA expression was significantly decreased in birds exposed to Pb (Figure 7).

## 4. Discussion

Pb has been shown to induce ROS generation, including hydroperoxides, singlet oxygen, and hydrogen peroxide (H_2_O_2_) [39]. Pb has also been shown to both elevate and suppress levels of SOD, CAT, and glutathione peroxidase (GPx) [40, 41]. Lower levels of exposure increase these enzymes, while higher exposure levels over long periods of time suppress them. In current study, as we used a moderate level of Pb exposure, SOD and CAT mRNA expression increased in response to Pb-induced oxidative stress. This is in line with previous studies and is related to the H_2_O_2_ accumulation and following lipid peroxidation [10, 42–44]. However, we observed a likewise change for GST*α* expression. Maintenance of the cellular glutathione (GSH) in different cellular compartments is also critically regulated by GSTs [45, 46]. The probable reason behind such observation is the positive correlation of GST*α* with Hsp70 expression. Studies by Volrn et al. [47] and Katsuki et al. [48] showed an interesting trend, in which cells under oxidative stress and tumors with high GSTs expression showed high Hsp70. In addition, immune-inhibition and immune-depletion studies showed that the Hsp70 chaperone is required for the efficient translation of GST*α* as well as its translocation to mitochondria [46, 49]. Hsp70 binding to GST*α* prevents its rapid dimerization in the cytosol, making it import competent to mitochondria. In other words, lack of Hsp70 or its downregulation affects GST*α* expression. This phenomenon is interestingly observed in our study, whereby GST*α* and Hsp70 expression are suppressed alongside in birds under Pb-induced oxidative stress. However, the question remains to be answered why Hsp70 expression is reduced in Pb-exposed birds? A possible explanation may be related to the upstream transcriptional regulation of Hsp70 expression via heat shock factor 1 (HSF1). Release of the HSF1 from the chaperone complex is ATP dependent and in turn is affected by general energy status of the cell [50, 51]. Therefore, it has been suggested that a critical ATP level is required for activation of HSF1 [52]. Study by Chang et al. [52] indicated that a moderate decrease in intracellular ATP correlates with activation of HSF1 and a severe depletion in ATP correlates with an attenuation in HSF1 activation. Accordingly in our study, significant suppression of feed intake and the shortage of energy and ATP supply may attenuate HSF1 activation and consequently reduced Hsp70 expression.

Our results showed that addition of 200 mg Pb/kg diet decreased feed intake and growth rate in broiler chicks. The decrease in feed intake and consequent growth rate suppression are difficult to explain with this study. However, recently, it has been speculated that Pb-induced decrease in brain 5-hydroxytryptamine (5-HT; serotonin) level upregulates 5-HT_2C_ receptor producing anorexia and anxiety in rats [53]. Furthermore, our result showed that Pb-induced oxidative stress negatively affected the feed efficiency. While this effect is in part attributable to the Pb-induced decrease in feed intake, it may also be directly related to associate metabolic and endocrine responses, as indicated by paired feeding studies [27, 54, 55]. Bolek and Persia [55] used a heat stress model in chicken and reported that approximately 54% of the reduced BWG was due to the effects of stress itself and the remaining BWG reduction was associated with reduced feed intake. It has been shown that chickens under heat stress have lower plasma thyroid hormones, weight, and length of the jejunum and higher corticosterone and apical SGLT1 expression than pair-fed groups [27]. At present, there is no report on the specific effect of Pb-induced oxidative stress in a pair-fed experiment. However, with regard to the definition of stress response as the “general nonspecific increase in arousal” [56], it is likely to expect the same phenomenon in Pb-induced oxidative stress. In this perspective, Pb stress-like response results in plasma corticosterone elevation [53, 57]. As a consequence, a vast range of glucocorticoids effects from decreasing feed intake to reducing intestinal absorptive surface area will take place [58]. In a physiological compensation feedback to maximize the sugar and amino acid uptake, nutrient transporters mRNA expression will be upregulated [27, 58–61]. In study by Garriga et al. [27], this response is reported to be triggered independently from the feed intake. They compared the SGLT1 activity and expression in heat stressed chickens with birds kept in thermoneutral condition and fed the same amount with heat stressed group. They observed that the activity and expression of SGLT-1 increased by ~50% in the heat stressed chickens, without effects in the pair-fed thermoneutral group, indicating that other signals may be involved in the heat stress response. A probable candidate to mediate these effects is corticosterone. Dexamethasone induces expression of SGLT-1 in various species small intestine [58, 60, 61]. However, Douard et al. [60] reported that glucocorticoids had no or modest effect on SGLT1-1 and GLUT2 expression. In disagreement, our results showed that Pb-exposure decreased the mRNA expression of SGLT1, SGLT4, GLUT2, PepT1, and EAAT3 in small intestine. Although there is no clear explanation for this observation, the discrepancies could be associated with the type of stressors. In Douard et al.'s [60] study, the stress condition was mimicked by glucocorticoid administration, while in our study we used Pb-induced oxidative stress model. Moreover, a close look at our data reveals an interesting phenomenon which, by Pb-induced oxidative stress, unlike the other sugar transporters, upregulated GLUT5 mRNA expression. This may be attributed to different functions of these sugar transporters. GLUT5 facilitates entry of fructose to epithelial cells through by facilitated diffusion. GLUT2 mediates the exit of monosaccharides from the enterocytes and SGLT1 and SGLT4 are responsible for the uptake of monosaccharide [35, 62, 63]. Pb exposure reduces entry of monosaccharides to enterocyte by downregulation of SGLT1 and SGLT4 and consequently reduces availability of GLUT2 substrate which in turn reduces mRNA expression of GLUT2. In response to this disruption and lack of energy in enterocyte level, the epithelial cells shift from energy consuming transporters (SGLT1 and SGLT4) to the one with no ATP requirement such as GLUT5 that is used to facilitate diffusion for fructose transportation. This speculation is supported by previous report of Douard et al. [60] where they showed that oxidative stress mimicked by dexamethasone injection dramatically increased expression of GLUT5 but not that of SGLT1 and GLUT2 in rat pups. Their investigation revealed six potential GR response elements in GLUT5 promoter regions 0–1250 bp upstream from the transcription start site, suggesting that dexamethasone could have a direct nuclear action. Therefore, it seems that GLUT5 regulation by glucocorticoids is specific and markedly different from SGLT1 and GLUT2.

In conclusion, our data showed that Pb-induced oxidative stress adversely suppresses feed efficiency and growth performance in chicken. The proposed underlying mechanism for such phenomenon is downregulation of major nutrient transporters genes in small intestine.

## Figures and Tables

**Table 1:** Ingredient composition of basal diet.

Ingredient (g/kg unless otherwise stated)	1–21 days
Ground yellow corn	538.9
Soybean meal	361.9
Fish meal	30.0
Palm oil	37.4
Choline chloride (60%)	2.5
Trimix^1^	1.0
Salt (NaCl)	2.0
DL-methionine	1.8
Limestone	13.0
Dicalcium phosphate	11.5
Total	**1000.0**
Calculated analysis (g/kg except energy)	
Crude protein	220.0
Crude fat	63.1
Crude fiber	38.0
Calcium	10.2
Phosphorus	4.5
Metabolisable energy (MJ/kg)	13.06

^1^Trimix (per kg Trimix): iron 100 g; manganese 110 g; copper 20 g; zinc 100 g; iodine 2 g; selenite 0.2 g; cobalt 0.6 g; santoquin 0.6 g; folic acid 0.33 g; thiamin 0.83 g; pyridoxine 1.33 g; biotin 2% 0.03 g; riboflavin 2 g; cyanocobalamin 0.03 g; D-calcium pantothenate 3.75 g; niacin 23.3 g; retinol 2000 mg; cholecalciferol 25 mg; *α*-tocopherol 23,000 mg IU.

**Table 2:** Primer sequences (5′ → 3′) used in real-time PCR.

Name	Forward primer	Reverse primer	Product size (bp)	Annealing Temperature °C
SOD^†^	AGGGGGTCATCCACTTCC	CCCATTTGTGTTGTCTCCAA	122	62.1
CAT	GGGGAGCTGTTTACTGCAAG	TTTCCATTGGCTATGGCATT	139	60
GST*α*	GCCTGACTTCAGTCCTTGGT	CCACCGAATTGACTCCATCT	131	62.1
Hsp70	AACCGCACCACACCCAGCTATG	CTGGGAGTCGTTGAAGTAAGCG	359	65
IL2	TGCAGTGTTACCTGGGAGAA	CTTGCATTCACTTCCGGTGT	135	60.2
IL6	GACTCGTCCGGAGAGGTTG	CGCACACGGTGAACTTCTT	128	60.2
SGLT1	TGTCTCTCTGGCAAGAACATGTC	GGGCAAGAGCTTCAGGTATCC	229	60.2
SGLT5	ATACCCAAGGTAATAGTCCCAAAC	TGGGTCCCTGAACAAATGAAA	75	60
GLUT2	CACACTATGGGCGCATGCT	ATTGTCCCTGGAGGTGTTGGTG	116	60
GLUT5	TTGCTGGCTTTGGGTTGTG	GGAGGTTGAGGGCCAAAGTC	99	60
PepT1	CCCCTGAGGAGGATCACTGTT	CAAAAGAGCAGCAGCAACGA	205	60
EAAT3	TGCTGCTTTGGATTCCAGTGT	AGCAATGACTGTAGTGCAGAAGTAATATATG	79	60
GAPDH	GCCGTCCTCTCTGGCAAAG	TGTAAACCATGTAGTTCAGATCGATGA	128	60
B-actin	ATGAAGCCCAGAGCAAAAGA	GGGGTGTTGAAGGTCTCAAA	175	60

^†^SOD (superoxide dismutase); CAT (catalase); GST-*α* (3-glutathione S-transferase-*α*); Hsp70 (heat shock protein); IL2 (interleukin 2); IL6 (interleukin 6); SGLT1 (Na^+^-dependent glucose and galactose transporter); SGLT5 (glucose transporter); GLUT2 (Na^+^-independent glucose, galactose and fructose transporter); GLUT5 (Na^+^-independent fructose transporter); PepT1 (oligopeptide transporter); EAAT3 (excitatory amino acid transporter 3, Na^+^, H^+^, K^+^ dependent); GAPDH (glyceraldehyde phosphate dehydrogenase).

## Abbreviations

Pb: Lead
PCR: Polymerase chain reaction
SGLT1: Na^+^-dependent glucose and galactose transporter
SGLT5: Glucose transporter
GLUT2: Na^+^-independent glucose, galactose, and fructose transporter
GLUT5: Na^+^-independent fructose transporter
PepT1: Oligopeptide transporter
EAAT3: Excitatory amino acid transporter
ALAD: Delta-aminolevulinic acid dehydratase
GAPDH: Glyceraldehyde phosphate dehydrogenase
FI: Feed intake
BWG: Body weight gain
Hsp70: Heat shock protein 70
5-HT: 5-Hydroxytryptamine
ROS: Reactive oxygen species
GSH: Glutathione
GST: Glutathione S-transferases
HSF: Heat shock factor
SOD: Superoxide dismutase
CAT: Catalase
Cd: Cadmium.
